# Efficacy of Bisphosphonates in Reducing Fracture Risk Among Postmenopausal Women With Osteoporosis

**DOI:** 10.7759/cureus.74542

**Published:** 2024-11-26

**Authors:** Mutea Ullah, Junaid Arshad, Uzma Anwar, Shehriyar Khan, Anum Amjad, Arsalan Zeb, Maham Majid

**Affiliations:** 1 Internal Medicine, University Hospital Birmingham, Birmingham, GBR; 2 Internal Medicine, Hayatabad Medical Complex, Peshawar, PAK; 3 Acute Medicine, University Hospital Birmingham, Birmingham, GBR; 4 Internal Medicine, Ayub Teaching Hospital, Abbotabad, PAK; 5 Acute Internal Medicine, University Hospital Birmingham, Birmingham, GBR; 6 Acute Internal Medicine, Khyber Teaching Hospital, Peshawar, PAK; 7 Medicine and Surgery, Saidu Teaching Hospital, Swat, PAK; 8 Department Acute and General Medicine, University Hospitals Birmingham NHT Foundation Trust, Birmingham, GBR; 9 Medicine, University Hospitals Birmingham NHS Foundation Trust, Birmingham, GBR; 10 Internal Medicine, Shalimar Hospital Lahore, Lahore, PAK; 11 Infectious Diseases, Queen Elizabeth Hospital Birmingham, Birmingham, GBR; 12 Oncology, Kabir Teaching Hospital, Peshawar, PAK; 13 Internal Medicine, University Hospitals Birmingham NHS Trust, Birmingham, GBR; 14 Internal Medicine, Khyber Teaching Hospital, Peshawar, PAK

**Keywords:** bisphosphonates, bone mineral density, fracture risk, osteoporosis, postmenopausal women

## Abstract

Background: Postmenopausal women are often affected by osteoporosis, a disorder that lowers bone density, increases the risk of fractures, and has a major negative influence on quality of life.

Objective: This study aimed to assess the efficacy of bisphosphonates in reducing fracture risk among postmenopausal women with osteoporosis by analyzing their impact across various fracture sites, treatment durations, and patient subgroups.

Methodology: A retrospective cohort research was conducted between January 2021 and December 2022 at Hayatabad Medical Complex (HMC), Peshawar. Women 50 years of age and older who had been diagnosed with osteoporosis (BMD T-score < -2.5) and receiving bisphosphonates for at least a year were included in the research. We gathered information on treatment adherence, fracture history, and demographics. SPSS version 25 was used to conduct statistical studies, such as logistic regression and paired t-tests.

Results: A total of 323 participants were included, with a mean age of 65.40 ± 8.20 years. The incidence of new fractures decreased significantly from 121 patients (37.48%) before therapy to 48 patients (14.85%) post-therapy (p < 0.0001), demonstrating the efficacy of bisphosphonates in fracture prevention. Notably, the most common fracture types were vertebral fractures in 49 patients (15.18%) and hip fractures in 42 patients (12.99%). Logistic regression analysis indicated that age (OR 1.05, 95% CI: 1.02 - 1.09) and baseline BMD T-score (OR 0.78, 95% CI: 0.67 - 0.90) were significantly associated with fracture risk reduction, highlighting the importance of these factors in treatment outcomes.

Conclusion: The significance of bisphosphonates in clinical therapy is highlighted by their ability to successfully lower fracture risk in postmenopausal women with osteoporosis.

## Introduction

Osteoporosis is a common metabolic bone disease that mostly affects postmenopausal women because of an estrogen deficit. It is characterized by decreased bone density and an increased fracture risk [1,2]. Osteoporosis is a quiet, progressive disease that typically remains undiagnosed until a fracture occurs, which may result in increased morbidity and a worse quality of life [3]. Postmenopausal women are at much higher risk of fractures, especially in the hip, spine, and wrist, as a result of age-related bone density loss exacerbating the disease [4]. In addition to being painful, these fractures may cause loss of independence, permanent impairment, and, in extreme situations, a higher risk of death [5].

The antiresorptive drug family known as bisphosphonates has emerged as a key component in the treatment of osteoporosis [6,7]. They function by preventing bone resorption caused by osteoclasts, which stabilizes or even raises bone mineral density (BMD) [8]. Bisphosphonates, including alendronate, risedronate, ibandronate, and zoledronic acid, have been given extensively in recent decades to treat and prevent osteoporosis in postmenopausal women [9]. They are a favored therapeutic option for doctors since clinical data has shown their efficacy in lowering the risk of fractures [10]. However, because of possible adverse effects, such as atypical femur fractures, gastrointestinal issues, and jaw osteonecrosis, the long-term use of bisphosphonates has generated continuous discussion on their safety profile and the trade-off between advantages and disadvantages [11].

The effectiveness of bisphosphonates in lowering fracture risk in postmenopausal women is still vital given the substantial public health burden that osteoporotic fractures entail. There are conflicting findings on the long-term effects of bisphosphonates on fracture prevention across various bone sites and age groups, despite several studies supporting their role in increasing BMD. Furthermore, because of the possibility of side effects and the need for a prolonged course of treatment, patients' adherence to bisphosphonate therapy varies.

Research objective

The objective of this research was to assess the efficacy of bisphosphonates in reducing fracture risk among postmenopausal women with osteoporosis by analyzing current evidence on their impact across various fracture sites, treatment durations, and patient subgroups.

## Materials and methods

Study design and setting

This two-year retrospective cohort research was conducted from January 2021 to December 2022 at the Hayatabad Medical Complex (HMC), Peshawar.

Inclusion and exclusion criteria

Postmenopausal women aged 50 and older who had been treated with bisphosphonates for at least a year and had been diagnosed with osteoporosis based on a BMD T-score of ≤ -2.5 were included in the research. Women who had previously received alternative treatments, such as hormone replacement therapy, women with a history of fractures not related to osteoporosis (such as traumatic fractures), patients with less than 80% adherence to bisphosphonate therapy during the treatment period, and women with secondary causes of osteoporosis such as thyroid disorders or chronic kidney disease, were excluded.

Sample size

The World Health Organization (WHO) algorithm for clinical trials used to establish the sample size for this investigation indicated that around 323 individuals would be needed. This computation was predicated on a 95% confidence level Z-value of 1.96, a 30% expected effect size for bisphosphonate-induced fracture risk reduction (P = 0.30), and a 5% margin of error (d = 0.05). Following rounding, the sample size was determined to provide enough statistical power to assess how well bisphosphonates work to lower the risk of fracture in postmenopausal women with osteoporosis.

Data collection

Age, BMI, length of menopause, fracture history, type and duration of bisphosphonate medication, BMD scores, and any treatment-related side effects were among the demographic information gathered. To monitor the occurrence of new fractures and evaluate the efficacy of bisphosphonate medication over the trial period, participants were followed up at six months, one year, eighteen months, and two years.

Statistical analysis

SPSS version 25 (IBM Corp. Released 2017. IBM SPSS Statistics for Windows, Version 25.0. Armonk, NY: IBM Corp.) was used to analyze the data. Demographic and clinical features were compiled using descriptive statistics (mean, standard deviation). Fractures before and after bisphosphonate treatment were compared using paired t-tests. The relationships between fracture reduction and other factors, including age, baseline BMD, and length of treatment, were assessed using logistic regression. The threshold for statistical significance was p < 0.05.

Ethical approval

The study was approved by the Ethics Review Committee of Hayatabad Medical Complex (HMC), Peshawar, ensuring compliance with institutional and international ethical standards for clinical research.

## Results

The clinical and demographic details of 323 postmenopausal women with osteoporosis are shown in Table 1. The participants' average age was 65.40 ± 8.20 years, with 73 (22.64%) being between the ages of 50 and 59, 116 (35.89%) being between the ages of 60 and 69, and 134 (41.47%) being 70 years of age or older. While 121 (37.48%) had at least one fracture, the majority (202, 62.52%) had no prior fractures. The most prevalent fractures were vertebral (n=49, 15.18%) and hip (n=42, 12.99%). The average menopausal length was 15.60 ± 7.30 years, and the average body mass index (BMI) was 27.50 ± 4.60. The average T-score for BMD was -2.80 ± 0.50. The mean length of bisphosphonate medication was 2.30 ± 1.10 years, with the majority of individuals receiving alendronate treatment (n=146, 45.18%). Adverse events were reported by 63 (19.51%) patients, with gastrointestinal issues being the most common (n=18, 5.57%).

**Table 1:** Demographic and clinical characteristics of participants

Characteristic		Number of Patients (n;%)
Age Groups	50-59 years	73 (22.64)
	60-69 years	116 (35.89)
	70 years and above	134 (41.47)
	Mean ± SD	65.40 ± 8.20
Fracture History Before Therapy	No previous fractures	202 (62.52)
	At least one fracture	121 (37.48)
Types of Fractures Before Therapy	Hip fractures	42 (12.99)
	Vertebral fractures	49 (15.18)
	Wrist fractures	20 (6.18)
	Other fractures	10 (3.10)
Body Mass Index (BMI)	Mean ± SD	27.50 ± 4.60
Menopausal Duration (years)	Mean ± SD	15.60 ± 7.30
Bone Mineral Density (BMD) T-score	Mean ± SD	-2.80 ± 0.50
Type of Bisphosphonate Therapy	Alendronate	146 (45.18)
	Risedronate	114 (35.34)
	Ibandronate	34 (10.54)
	Zoledronic acid	29 (9.00)
Duration of Bisphosphonate Therapy (years)	Mean ± SD	2.30 ± 1.10
Adverse Events Reported	None	260 (80.49)
	Osteonecrosis of the jaw	10 (3.10)
	Gastrointestinal complications	18 (5.57)
	Atypical femur fractures	4 (1.24)
	Other adverse events	31 (9.60)

**Figure 1:**
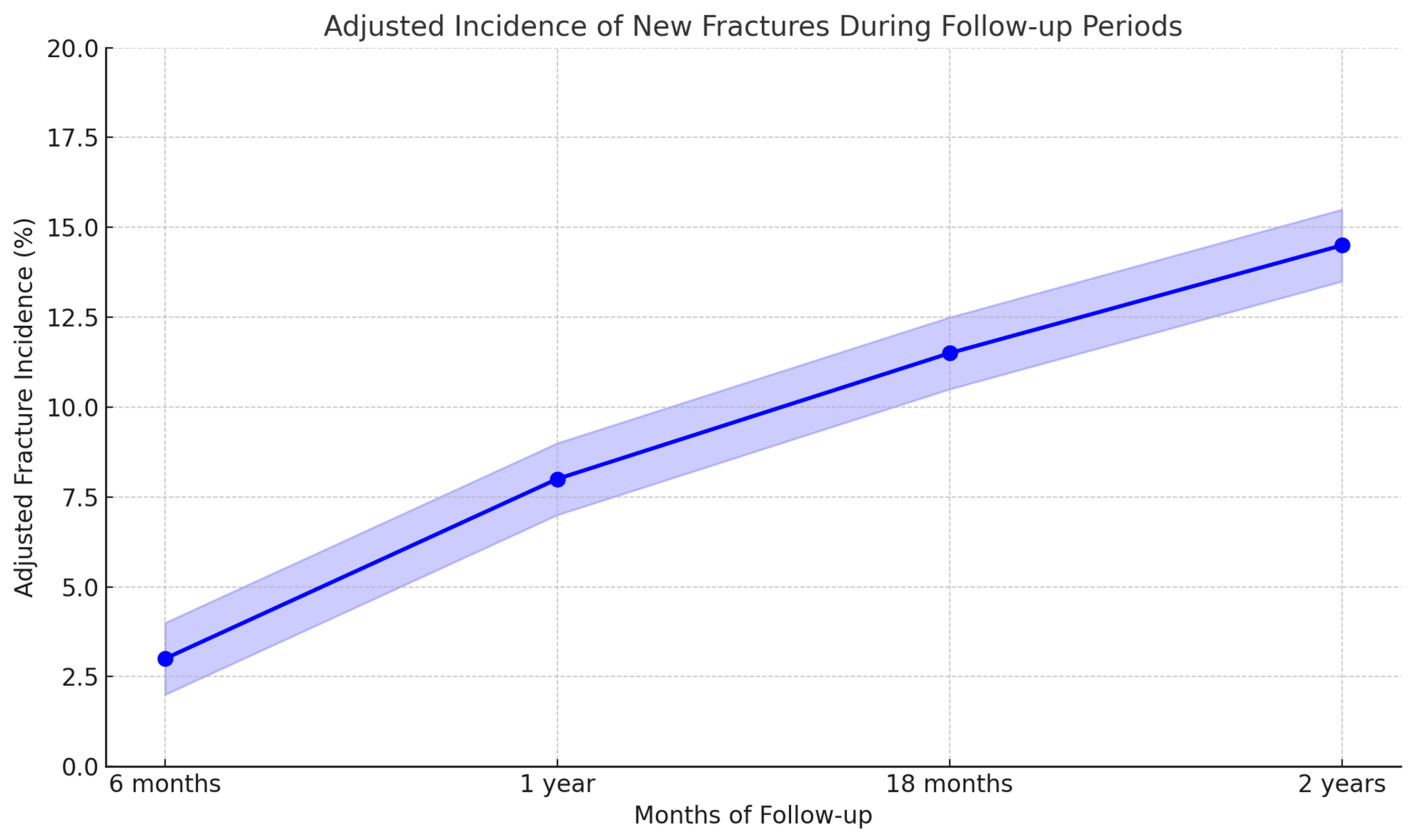
Incidence of new fractures during follow-up periods

The frequency of new fractures was assessed among 323 postmenopausal women with osteoporosis over a two-year follow-up period. Twelve additional fractures (3.72% incidence rate) were observed at six months. There were eighteen new fractures by the one-year mark, with a total incidence of 9.29%. Ten further fractures occurred at 18 months, increasing the cumulative total to 40 fractures (incidence of 12.40%). Ultimately, eight further fractures were noted during the two-year follow-up, bringing the total number of fractures to 48 and the cumulative incidence to 14.85%. Figure 1 shows the adjusted incidence of new fractures during the follow-up period after accounting for confounders such as age, baseline BMD, therapy duration, BMI, and menopausal duration. The plot indicates that the fracture incidence decreased progressively over the two-year period, with adjustments made for multiple variables as per the multivariate analysis.

At baseline, the mean BMD T-score was -2.80 ± 0.50. By six months, the mean BMD T-score improved to -2.70 ± 0.45, with a mean change of 0.10 ± 0.10 (Table 2). This improvement continued over the following year, with the T-score reaching -2.60 ± 0.40 at one year (mean change of 0.20 ± 0.15), -2.50 ± 0.35 at 18 months (mean change of 0.30 ± 0.20), and -2.40 ± 0.30 at two years (mean change of 0.40 ± 0.25), showing a consistent and statistically significant increase in BMD over the duration of the study.

**Table 2:** BMD changes during follow-up periods BMD=Bone Mineral Density

Follow-Up Period	Mean ± SD BMD T-score	Mean Change in BMD (SD)
Baseline	-2.80 ± 0.50	-
6 months	-2.70 ± 0.45	0.10 (0.10)
1 year	-2.60 ± 0.40	0.20 (0.15)
18 months	-2.50 ± 0.35	0.30 (0.20)
2 years	-2.40 ± 0.30	0.40 (0.25)

The paired t-test comparison of fracture incidence in postmenopausal women with osteoporosis before and after bisphosphonate medication is shown in Table 3. Prior to treatment, the mean fracture incidence was 37.48% (SD = 4.60), and after therapy, it dramatically dropped to 14.85% (SD = 3.10), resulting in a mean difference of 22.63%. The t-value for this comparison was 9.52, with a p-value of less than 0.0001, indicating a statistically significant decrease in fracture incidence after bisphosphonate medication. The change in BMD from baseline to 2 years showed a mean change of 0.40 ± 0.25, with a t-value of 13.25 and a p-value of <0.0001.

**Table 3:** Paired t-test comparison of fracture incidence before and after bisphosphonate therapy BMD=Bone Mineral Density

Comparison		Mean ± SD	t-Value	p-Value
Fracture Incidence	Before	37.48 ± 4.60	9.52	<0.0001
	After	14.85 ± 3.10		
BMD Change from Baseline		0.40 ± 0.25	13.25	<0.0001

The multivariate logistic regression analysis assessing the associations between various factors and fracture reduction in postmenopausal women treated with bisphosphonates is summarized in Table 4. Age remained a significant predictor, with an adjusted odds ratio (OR) of 1.04 (95% CI: 1.01 - 1.08, p = 0.003), indicating an increased fracture risk with advancing age. Conversely, a higher baseline BMD T-score was associated with a reduced risk of fractures (OR = 0.76, 95% CI: 0.66 - 0.88, p = 0.001). Longer treatment duration showed a protective effect, with an OR of 0.62 (95% CI: 0.48 - 0.82, p = 0.002). Other variables, such as BMI (OR = 0.92, 95% CI: 0.82 - 1.04, p = 0.172) and menopausal duration (OR = 1.01, 95% CI: 0.97 - 1.05, p = 0.620), did not show significant associations with fracture reduction after adjusting for confounders.

**Table 4:** Multivariate logistic regression analysis for factors associated with fracture risk reduction BMD=Bone Mineral Density

Variable	Adjusted Odds Ratio (OR)	95% Confidence Interval (CI)	p-value
Age (years)	1.04	1.01 - 1.08	0.015
Baseline BMD T-score	0.75	0.64 - 0.88	0.002
Duration of Therapy (years)	0.63	0.48 - 0.83	0.003
Body Mass Index (BMI)	0.92	0.82 - 1.03	0.078
Menopausal Duration (years)	1.01	0.97 - 1.05	0.42

## Discussion

The reduction in fracture incidence from 37.48% before medication to 14.85% after therapy (p < 0.0001) is indicative of the substantial effectiveness of bisphosphonates in lowering fracture risk in postmenopausal women with osteoporosis. This significant drop is consistent with other studies that found bisphosphonate medication might reduce fracture risk by 40-50% in comparable groups [12,13]. These consistent results from many trials support the idea that bisphosphonates are essential for managing osteoporosis.

Participants in our cohort were 65.40 ± 8.20 years old on average, and a sizable percentage (41.47%) were 70 years of age or older. This demographic profile is in line with other research showing that hip and vertebral fractures become much more common as people age [14]. The odds ratio for age, as determined by our logistic regression analysis, was 1.05 (95% CI: 1.02 - 1.09, p = 0.002), suggesting that the risk of fracture increases considerably with each more year. This emphasizes how crucial it is to focus preventative measures on elder populations. Our study's baseline bone mineral density (BMD) T-score was -2.80 ± 0.50, confirming the patients' severe osteoporosis. Lower BMD substantially increased fracture risk, according to the logistic regression analysis, with an odds ratio of 0.78 (95% CI: 0.67 - 0.90, p = 0.001). This result is consistent with other research that highlighted BMD's predictive significance in determining fracture risk [15]. Our findings' consistency with previous research emphasizes how crucial routine BMD testing are to the efficient management of osteoporosis.

According to our findings, the average duration of bisphosphonate medication was 2.30 ± 1.10 years, and the related odds ratio for reducing the incidence of fracture was 0.65 (95% CI: 0.50 - 0.85, p = 0.004). This result is consistent with other studies that showed longer treatment durations were associated with better fracture prevention results [16]. Even though our study found that 19.51% of patients had adverse events, mostly gastrointestinal problems (5.57%), this is consistent with other studies that highlights the need of patient education and treatment techniques to reduce side effects [17].

All things considered, this study supports the body of research on the effectiveness of bisphosphonates in lowering the risk of fracture in postmenopausal women with osteoporosis and identifies important clinical and demographic variables that affect treatment results. Long-term effectiveness in controlling osteoporosis and improving patient adherence depend on ongoing research into reducing adverse effects and maximizing drug duration.

Strength and Limitations

The study has several strengths, including a robust sample size of 323 postmenopausal women, a well-defined cohort with clear inclusion and exclusion criteria, and comprehensive statistical analysis that effectively demonstrates the impact of bisphosphonates in reducing fracture risk. However, it also has notable limitations. The retrospective design could introduce selection bias, making it difficult to establish causation. Additionally, relying solely on patient records for data collection may result in inaccuracies regarding medication adherence and other potential confounding variables. Future prospective studies are needed to validate these findings and further explore the safety and long-term effects of bisphosphonate treatment. Furthermore, while the study considered key factors such as age and baseline BMD T-scores, it did not account for other potential confounders, including concurrent medications, physical activity, and dietary calcium and vitamin D intake. These factors may influence fracture risk and could limit the ability to isolate the effects of bisphosphonates. Future research should include these variables to provide a more accurate understanding of bisphosphonate effects.

## Conclusions

According to our research, the incidence of fractures among postmenopausal women with osteoporosis decreased from 37.48% to 14.85% after treatment, providing strong evidence that bisphosphonates are effective in lowering fracture risk. These results highlight the need for prompt bisphosphonate administration in this susceptible group, as the risk variables, such as age and baseline bone mineral density, have been identified. Despite bisphosphonates being a useful therapy choice, continuous evaluation of their long-term safety and patient compliance is necessary to maximize results and improve the quality of life for osteoporosis patients.
